# Wernicke's encephalopathy: Is visual loss a red herring?

**DOI:** 10.1002/ccr3.6372

**Published:** 2022-09-25

**Authors:** Sujit Kumar, Abdul Rawoof Bolar, Rohit Shetty, Sharath Kumar Goddu Govindappa, Manithody Narayan Bhat Pramod, Jagadish Basavaraj Agadi, Lakshminarayanapuram Gopal Vishwanathan, Chaitra Parameshwara Adiga

**Affiliations:** ^1^ Apollo Hospitals Bangalore India; ^2^ Comprehensive and Neuroopthalmology Narayana Nethralaya Bangalore India; ^3^ Narayan Nethralaya Bangalore India; ^4^ Apollo Hospitals Bangalore India; ^5^ Department of Neurology Apollo Hospitals Bangalore India; ^6^ Apollo Hospitals Bangalore India

**Keywords:** hyperemesis gravidarum, ophthalmoparesis, papilloedema, retinal hemorrhages, thiamine

## Abstract

Predominantly visual loss, is very rare in Wernicke's encephalopathy. A 22 year old lady, in her 28th week of gestation, presented with a confused mental state, bilateral papilloedema with retinal hemorrhages, ophthalmoparesis, and cerebellar signs. Her MRI brain was suggestive of Wernicke's encephalopathy and she recovered with intravenous thiamine

## Graphical abstract

Visual loss as a presenting clinical feature of Wernicke's encephalopathy is very rare and must be considered in an appropriate clinical setting, such as hyperemesis gravidarum, in order to facilitate early treatment. Visual loss, characterized by retinal hemorrhages and disc edema, may be reversible in Wernicke's encephalopathy if treated early with Thiamine.

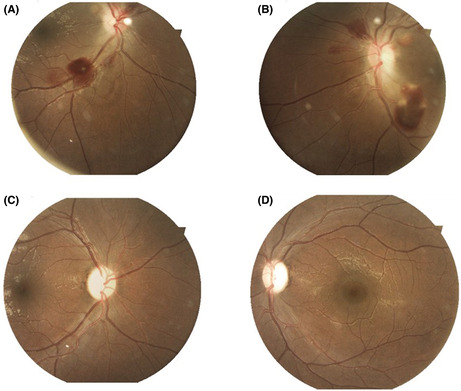

## 1 INTRODUCTION

“Polioencephalitis hemorrhagica superioris” was the original term coined by Carl Wernicke in 1881 to describe the classical syndrome, with a triad of ophthalmoparesis, ataxia, and mental status changes. Typically, it is encountered in clinical situations involving poor nutrition such as alcoholics, post gastrointestinal surgery, hyperemesis gravidarum, malignancy, and other such states.^1^ Operational criteria proposed for Wernicke's encephalopathy need any two of the following: Dietary inadequacies, eye movement abnormalities, ataxia and either an altered mental state or memory disturbances.^2^ Thiamine is the mainstay of treatment. Although in the initial series of Adams, 100 mg per day was recommended, many subsequent studies recommend 500 mg, initially, parenterally.^3^, ^4^

Visual loss is an unusual and rare manifestation of Wernicke's encephalopathy. In the original series of Adams et al., only 3 of 232 patients had retinal hemorrhages with visual loss.^4^ Papilloedema with Wernicke's encephalopathy has been described only rarely in a few case reports. We describe a patient of Wernicke's, who presented with predominantly visual loss.

## 2 CASE REPORT

A 22 year old lady presented with a history of blurring of vision since 6 days. The visual blurring was acute at onset and became progressive over 3 days. She was 28 weeks pregnant and had a history of vomiting since 3 months. Clinical examination revealed that, although conscious, she was confused and not oriented to time, place or person. Her bilateral corrected visual acuity was 6/9 bilaterally. There was a bilateral sixth nerve palsy. She was able to stand with minimal support and had stance/ gait ataxia. Further, there were bilateral cerebellar signs on examination.

**FIGURE 1:**
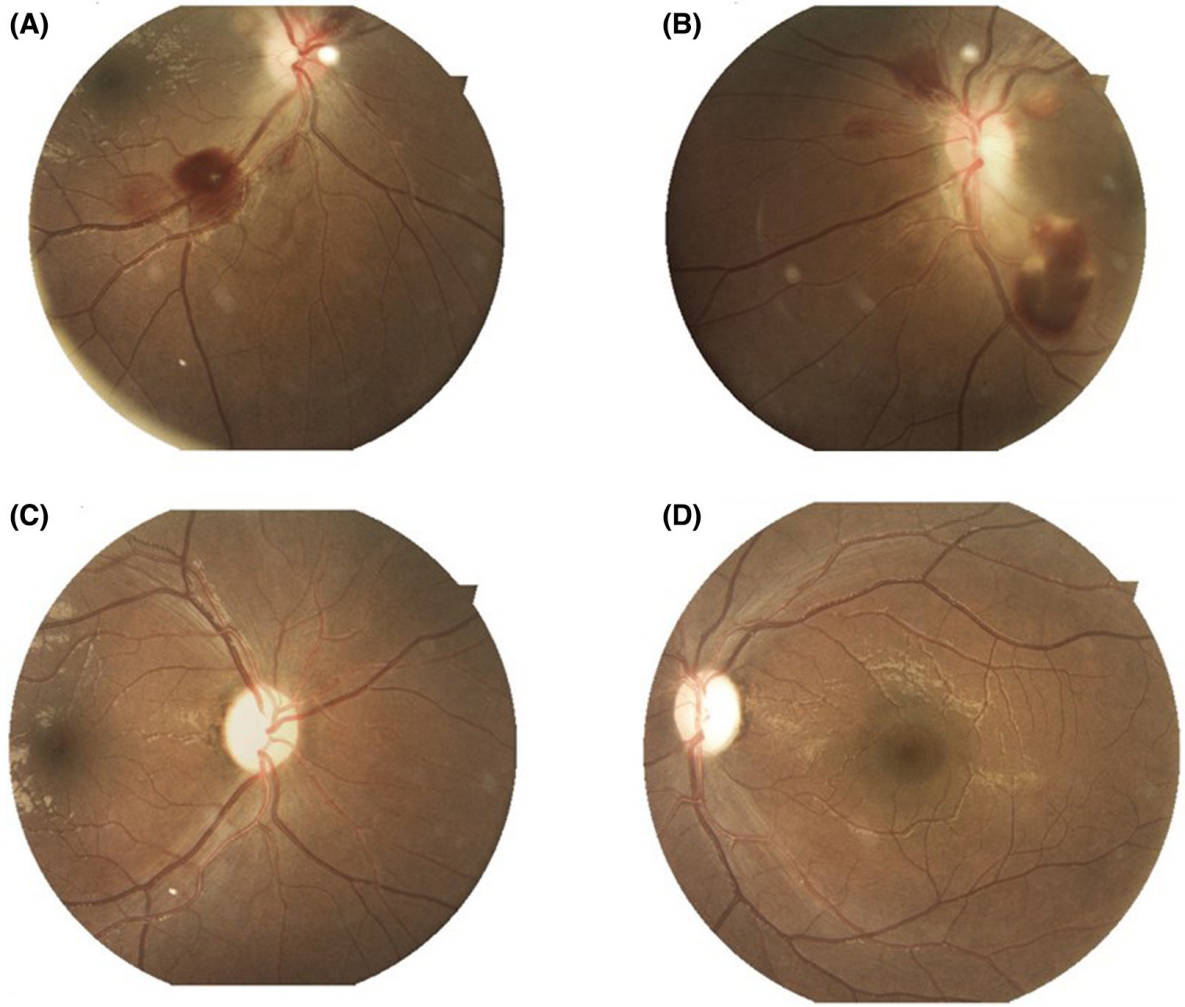
(A) and (B) show disc edema in the right and left eyes respectively, with retinal hemorrhages. (C) and (D) show resolution of disc edema, after treatment with thiamine in the right and left eyes respectively with disc pallor.

Fundoscopy revealed bilateral disc edema with retinal hemorrhages (Figure 1A,B). Her blood investigations revealed a sodium level of 128 meq/l, and a potassium level of 2.9 meq/l, and she tested positive for urine ketone bodies. An echocardiogram was performed, which revealed normal cardiac function. A nerve conduction study of all four limbs was done, which was normal.

**FIGURE 2:**
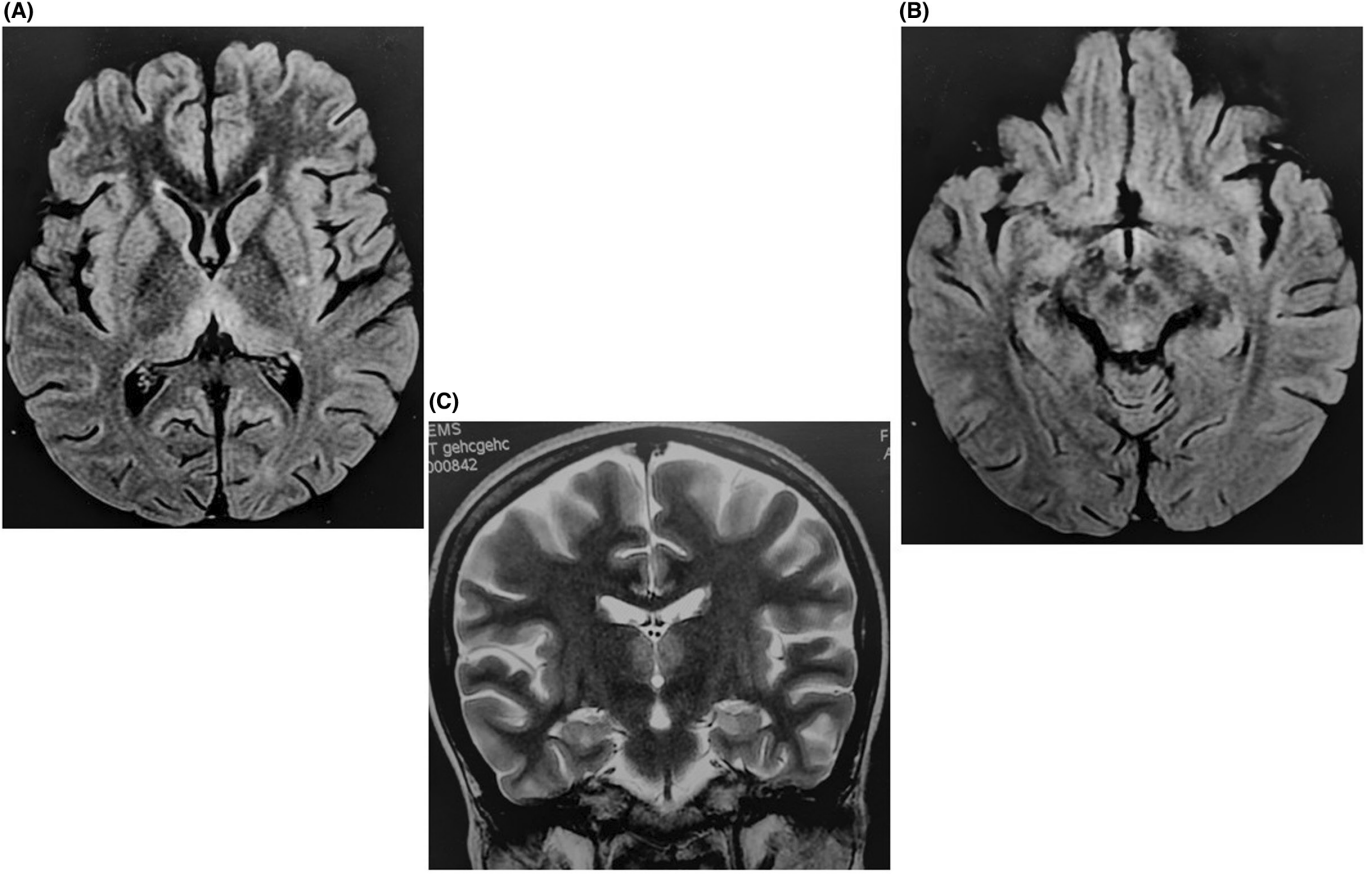
(A) showing hyperintensities on FLAIR images in bilateral medial thalami. (B) showing hyperintensities on FLAIR images in periaqueductal gray matter. (C) showing hyperintensities in bilateral mamillary bodies.

**FIGURE 3:**
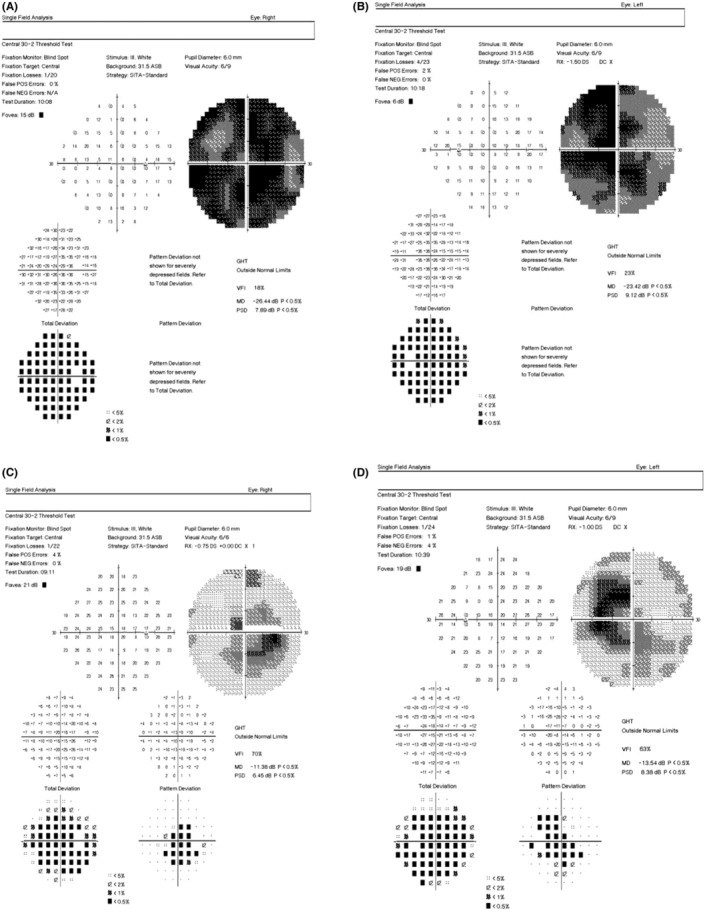
(A) and (B) represent visual fields of right and left eyes respectively with severe field constriction. (C) and (D) represent visual fields of right and left eyes respectively, a month after treatment with centrocecal scotomas.

MRI brain scan was done and the results showed Fluid attenuated inversion recovery (FLAIR) hyperintensities in bilateral medial thalami, periaqueductal gray matter, and mamillary bodies (Figure 2). Visual fields showed severe constriction bilaterally (Figure 3A,B: Right and left eyes, respectively). Thus, a diagnosis of Wernicke's encephalopathy was made.

The patient was treated with thiamine parenterally, with 500 mg iv on the first day, followed by 100 mg intravenously thrice a day for 7 days. Her vision improved dramatically with the treatment. The bilateral sixth nerve palsy and cerebellar signs also resolved in 1 week. She was continued on oral thiamine 100 mg twice daily for a month.

The disc edema resolved completely in 1 month (Figure 1C,D) and visual fields improved too (Figure 3C,D, Right and Left eyes respectively).

## 3 DISCUSSION

Wernicke's encephalopathy is a serious nutritional disorder with potentially life‐ threatening consequences, if unrecognized. Although, the triad of opthalmoparesis, changes in higher mental functions and ataxia are easily recognized, there may be confounding clinical features such as blurring of vision, which is rarely seen, thus delaying the diagnosis. Among the ocular findings, nystagmus is most common, followed by bilateral 6th nerve paresis, conjugate gaze palsy, scotomata, pupillary abnormalities, retinal hemorrhages, ptosis, and disc edema.^5^

Disc edema is a rare finding in Wernicke's encephalopathy.^6^ Retinal hemorrhages are seen more frequently, without disc edema. These manifestations have been described with bariatric surgery too. In the patients described, disc edema with retinal hemorrhages accompanied the vision loss, along with other characteristic manifestations. The patients showed a good response to intravenous thiamine.^7^, ^8^

Mumford described a similar case of disc edema with retinal hemorrhages in a case of hyperemesis gravidarum, with other features of Wernicke's encephalopathy, who responded well to parenteral thiamine administration.^1^

Chitra et al.^9^ described a 25 year old lady in her 20th week of pregnancy, who presented with visual loss, disc edema, retinal hemorrhages, ataxia, and ophthalmoparesis. The clinical features improved rapidly with parenteral thiamine.

Thiamine prophylaxis in patients with hyperemesis gravidarum has also been recommended, starting from 50 mg per day to 200 to 1200 mg per day parenterally for 2 weeks followed by oral thiamine of 60 mg per day till the end of gestation.^10^

The initial treatment dose of 500 mg thiamine three times daily has been recommended by the Royal college of physicians and European federation of neurological societies, which is believed to result in a quick resolution of neurological symptoms.^11^

Visual loss as a presenting clinical feature of Wernicke's encephalopathy is very rare and must be considered in an appropriate clinical setting, in order to facilitate early treatment.

The mechanisms underlying the visual manifestations are not clear. It is likely that an optic neuropathy associated with Wernicke's encephalopathy, due to nutritional deficiency of thiamine could result in the disc edema. This is consistent with quick resolution of fundoscopic findings with thiamine administration. Another plausible explanation is the possible necrosis of nerve cells with edema, which underlies neuropathology of Wernicke's.^1^

## 4 CONCLUSIONS

Early treatment with parenteral thiamine can prevent the cognitive complications of Wernicke's encephalopathy. Our case illustrates that, in a setting such as pregnancy with hyperemesis gravidarum, where there is an enhanced metabolic requirement of thiamine, one must have a high index of suspicion in patients presenting primarily with visual loss and other features of Wernicke's encephalopathy. This is especially important as disc edema and retinal hemorrhages are rare in Wernicke's encephalopathy. This would prevent a potential delay in diagnosis and facilitate urgent treatment with parenteral thiamine. Thus, visual loss may be reversible in Wernicke's encephalopathy, if treated early with thiamine.

## AUTHOR CONTRIBUTIONS

**Sujit Kumar**: Clinical management of the case and preparation of manuscript. **Abdul Rawoof Bolar**: Neuroopthalmological evaluation of the case and preparation of manuscript. **Rohit Shetty**: Neuroopthalmological evaluation of the case and preparation of manuscript. **Sharath Kumar Goddu Govindappa**: Neuroradiological assessment and preparation of manuscript. **Manithody Narayana Bhat Pramod**: Clinical management of the case and preparation of manuscript. **Jagadish Basavaraj Agadi**: Clinical management of the case and preparation of manuscript. **Lakshminarayanapuram Gopal Vishwanathan**: Clinical management of the case and preparation of manuscript. **Chaitra Prameshwara Adiga**: Neuroradiological assessment and preparation of manuscript.

## FUNDING INFORMATION

No funding from any source was used for this study.

## CONFLICT OF INTEREST

The authors have no conflict of interest to disclose.

## CONSENT

A written consent has been obtained from the patient. It can be provided if requested.

## Data Availability

Data available on request from the authors

## References

[1] Mumford CJ . Papilloedema delaying diagnosis of Wernicke's encephalopathy in a comatose patient. Postgrad Med J. 1989;65:371‐373. 2608577 10.1136/pgmj.65.764.371 PMC2429353

[2] Caine D , Halliday GM , Kril JJ , Harper CG . Operational criteria for the classification of chronic alcoholics: identification of Wemicke's encephalopathy. J Neurol Neurosurg Psychiatry. 1997;62:51‐60. 9010400 10.1136/jnnp.62.1.51 PMC486695

[3] Chataway J , Hardman E . Thiamine in Wernicke's syndrome—how much and how long? Postgrad Med J. 1995;71:249. 10.1136/pgmj.71.834.249 PMC2398086 7784292

[4] Victor M , Adams RD , Collins GH . The Wernicke‐Korsakoff Syndrome and Related Neurologic Disorders Due to Alcoholism and Malnutrition. 2nd ed. FA Davis; 1989.

[5] Victor M , Adams RD , Collins GH . The Wernicke‐Korsakoff syndrome. A clinical and pathological study of 245 patients, 82 with post‐mortem examinations. Contemp Neurol Ser. 1971;7:1‐206. 5162155

[6] Reuler JB , Girard DE , Cooney TG . Current concepts. Wernicke's encephalopathy. N Engl J Med. 1985;312:1035‐1039. 3885034 10.1056/NEJM198504183121606

[7] Lawton AW , Frisard NE . Visual loss, retinal hemorrhages, and optic disc edema resulting from thiamine deficiency following bariatric surgery complicated by prolonged vomiting. Ochsner J. 2017;17:112‐114. 28331457 PMC5349621

[8] Bohnsack BL , Patel SS . Peripapillary nerve fiber layer thickening, telangiectasia, and retinal hemorrhages in Wernicke Encephalopathy. J Neuroophthalmol. 2010;30:54‐58. 20182209 10.1097/WNO.0b013e3181ceb4d0

[9] Chitra S , Latha KVS . Wernicke's Encephalopathy with visual loss in a patient with hyperemesis gravidarum. J Assoc Physicians India. 2012;60:53‐56. 23029727

[10] Kotha VK , De Souza A . Wernicke's Encephalopathy following hyperemesis gravidarum, a report of three cases. Neuroradiol J. 2013;26(1):35‐40. 23859165 10.1177/197140091302600106 PMC5278861

[11] Oudman E , Wijnia JW , Dam MV , Postma A . Wernicke‐Korasakoff syndrome despite no alcohol use: a summary of systematic reports. J Neurol Sci. 2021;426:1‐6. 10.1016/j.jns.2021.117482 34000679

